# Utility of Ugandan genomic selection cassava breeding populations for prediction of cassava viral disease resistance and yield in West African clones

**DOI:** 10.3389/fpls.2022.1018156

**Published:** 2022-11-23

**Authors:** Alfred A. Ozimati, Williams Esuma, Francis Manze, Paula Iragaba, Michael Kanaabi, Chukwuka Ugochukwu Ano, Chiedozie Egesi, Robert S. Kawuki

**Affiliations:** ^1^ National Crops Resources Research Institute, Kampala, Uganda; ^2^ Department of Plant Sciences, Microbiology and Biotechnology, College of Natural Sciences, Makerere University, Kampala, Uganda; ^3^ Plant Breeding and Genetics Section, College of Agricultare and Life Sciences, Cornell University, Ithaca NY, United States; ^4^ National Root Crops Research Institute, Umudike, Nigeria; ^5^ International Institute of Tropical Agriculture (IITA), Ibadan, Nigeria

**Keywords:** cassava, genomic prediction, training population and viral diseases, models, validation set

## Abstract

Cassava (*Manihot esculenta* Crantz) is a staple crop for ~800 million people in sub-Saharan Africa. Its production and productivity are being heavily affected by the two viral diseases: cassava brown streak disease (CBSD) and cassava mosaic disease (CMD), impacting greatly on edible root yield. CBSD is currently endemic to central, eastern and southern Africa, if not contained could spread to West Africa the largest cassava producer and consumer in the continent. Genomic selection (GS) has been implemented in Ugandan cassava breeding for accelerated development of virus resistant and high yielding clones. This study leveraged available GS training data in Uganda for pre-emptive CBSD breeding in W. Africa alongside CMD and fresh root yield (FRW). First, we tracked genetic gain through the current three cycles of GS in Uganda. The mean genomic estimated breeding values (GEBVs), indicated general progress from initial cycle zero (C0) to cycle one (C1) and cycle two (C2) for CBSD traits and yield except for CMD. Secondly, we used foliar data of both CBSD and CMD, as well as harvest root necrosis and yield data to perform cross-validation predictions. Cross-validation prediction accuracies of five GS models were tested for each of the three GS cycles and West African (WA) germplasm as a test set. In all cases, cross-validation prediction accuracies were low to moderate, ranging from -0.16 to 0.68 for CBSD traits, -0.27 to 0.57 for CMD and -0.22 to 0.41 for fresh root weight (FRW). Overall, the highest prediction accuracies were recorded in C0 for all traits tested across models and the best performing model in cross-validation was G-BLUP. Lastly, we tested the predictive ability of the Ugandan training sets to predict CBSD in W. African clones. In general, the Ugandan training sets had low prediction accuracies for all traits across models in West African germplasm, varying from -0.18 to 0.1. Based on the findings of this study, the cassava breeding program in Uganda has made progress through application of GS for most target traits, but the utility of the training population for pre-emptive breeding in WA is limiting. In this case, efforts should be devoted to sharing Ugandan germplasm that possess resistance with the W. African breeding programs for hybridization to fully enable deployment of genomic selection as a pre-emptive CBSD breeding strategy in W. Africa.

## Introduction

The raising energy demand globally in face of climate change is popularizing cassava as an alternative source of renewable fuel with full potential to replace fossil fuel in the developed countries (Kang et al., 2014). Besides, being a potential crop to generate renewable fuel at global level, cassava is a major of source of carbohydrate and staple food for over 800 million people in the world (Hammond et al., 2013). Because of the global importance of the cassava, its production has steadily increased world-wide in last the two decades from 162 MT in 1998 to 303 MT in 2018 (FAO, 2019), with the world’s highest production of ~ 60 MT coming from Nigeria in W. Africa.

Despite the importance of cassava as food security, especially in sub-Saharan Africa, average yields still remain low (12 t/ha) compared with yield average of 20 t/ha recorded in Asia countries like Thailand (Nweke, 2004). A number of biotic and abiotic factors contribute to this yield gap in sub-Saharan Africa. The leading biotic stress being cassava brown steak disease (CBSD) and cassava mosaic disease (CMD) (Legg et al., 2014). While CMD is present in all cassava producing areas in Africa and Asia, CBSD is only endemic to Eastern and Southern and Central Africa and more recently the disease was reported in Angola, which is closer to West Africa especially Nigeria, the largest cassava producer and consumer in the world (Ano et al., 2021). In highly susceptible varieties, yield losses of up to 100% have been reported (Hillocks et al., 2002; Alicai et al., 2007). The recent epidemiological studies indicate that CBSD is fast spreading to West Africa (Patil et al., 2015), and thus posing an eminent threat to cassava production in the Western part of the continent.

Fortunately, cassava that lagged previously in terms of genomic resources relative to cereal crops like maize, rice and wheat and legumes such as common beans, ground nuts and soya beans, has received significant funding to develop complete reference genome assembly (Prochnik et al., 2012). With the availability of the genomic resources for cassava and low-cost genotyping technologies such as genotyping-by-sequencing (Elshire et al., 2011; Rabbi et al., 2015) and more recently the diversity array technology sequencing (DArTSeq) platform, cassava breeding is evolving from traditional phenotypic selection to selecting plants based on their genomic estimated breeding values (GEBVs). Genomic selection (GS), which uses high-density markers to cover the entire genome, was proposed by Meuwissen et al. (2001) as a new method for selection of individuals in a population based on the breeding values.

Genomic selection has been reported to offer some advantages over phenotypic selection breeding scheme: (i) genomic selection allows for more cycles of recombination per unit time than phenotypic selection, (ii) selection is solely based on estimates of marker effects without prior knowledge of the QTL and also captures variation due to loci with small effects (de Oliveira et al., 2012). Another argument put in favor of genomic selection is that genotyping cost will further decrease per sample; on the other hand, phenotyping costs do not exhibit the same downward trend, because they are dependent on human resources and agricultural inputs. The cost of these resources have historically been increasing (de Oliveira et al., 2012; Poland and Rife, 2012).

Through the Next Generation Cassava Breeding project, Uganda embraced genomic selection tool for cassava improvement in early 2010s. The breeding program has so far developed three recurrent genomic selection cycles, and some of the elite material has been channeled to the variety development pipeline. Our primary traits of focus include: CMD resistance, fresh root weight, end-user root quality attributes (Wolfe et al., 2017) and CBSD resistance. From historical data, Uganda has registered significant gains for CMD and CBSD resistance breeding efforts (Manze et al., 2021), and thus could offer CBSD resistant parents to west African cassava breeding programs such as Nigeria where CBSD is not yet a threat. However, because of the CBSD pandemic in Eastern, Southern and Central Africa, there is restriction on moving plant materials currently from Eastern, Central and Southern Africa to West Africa, where CBSD is non-existent (Ano et al., 2021). Nonetheless, illegal movement of plant materials due to porous borders, besides the whitefly supported transmission could pose a risk of CBSD reaching to West Africa (Legg et al., 2014).

We previously leveraged on the available genomic resources under Next Generation Cassava Breeding project (https://www.nextgencassava.org/) to predict CBSD in West Africa using 35 clones shared from IITA, Ibadan (Ozimati et al., 2018). Generally, low predictive ability, ranging from 0.14 to 0.36 for CBSD foliar severity, and -0.29 to 0.11 for CBSD root necrosis (Ozimati et al., 2018) were recorded. Building on previous CBSD pre-emptive breeding study, which was limited by the sample size, we expanded on sample size of the West Africa test set used in the current study. Specifically, we assessed gains from genomic selection for virus disease resistance and fresh root weight in Ugandan GS training populations, and further evaluated effectiveness of the training sets in predicting CBSD, CMD resistance and fresh root yield in WA clones as pre-emptive CBSD breeding strategy.

## Materials and Methods

### Germplasm and field evaluation

The training population comprised three recurrent genomic selection cycles obtained from NaCRRI. These cycles were: cycle zero (C0), cycle one (C1) and cycle two (C2). Briefly, C0 population was derived from forty-nine diverse progenitors that were assembled from International Institute of Tropical Agriculture (IITA), International Center for Tropical Agriculture (CIAT) and NaCRRI. Germplasm from CIAT (Columbia) targeted improvement of quality and yield traits, while germplasm from the IITA (Tanzania), and NaCRRI (Uganda) breeding programs targeted improvement of CBSD resistance. Botanical seeds from crosses (full-sibs and half-sibs) of forty-nine progenitors were planted in a seedling nursery at Namulonge, and the sprouted seedlings were evaluated in an unreplicated seedling trial at Namulonge in 2012. A total of 466 C0 seedlings were selected visually as a training population for implementation of GS based on their CMD and CBSD resistance, and evaluated for two years (2013 and 2014) at Namulonge (central Uganda), Kasese (mid-western Uganda) and Ngetta (northern Uganda), using an alpha lattice design with two replications. Namulonge, Kasese and Ngetta were specifically chosen because of high viral disease pressure (cassava brown streak disease and cassava mosaic disease) and whitefly (vector) populations (Alicai et al., 2019). The C1 clones were derived from recurrent selection and recombination of the best a hundred C0 clones selected through GS. A total of 667 C1 seedlings were selected visually and evaluated in a clonal trial that was laid out using an augmented randomized block design at both Namulonge and Serere in 2016 and 2017. Similarly, the top hundred performers selected from C1 clonal trial were recombined to generate the C2 population. The C2 clonal trial comprised 421 clones and was also laid out using an augmented randomized block design in 2019 at Namulonge for one season. Selection of progenitors for constitution of C1 and C2 were based on CMD resistance, CBSD resistance, harvest index and fresh root yield.

All clones in the training set (C0, C1 and C2) were evaluated for CBSD and CMD severity, fresh root weight and harvest index. CBSD foliar severity was assessed at three (CBSD3S) and six CBSD6S) months after planting using a standard scale of 1-5; where 1 = no apparent symptoms, 2 = slight foliar chlorosis, but with no stem lesions, 3 = pronounced foliar chlorosis and mild stem lesions with no die back, 4 = severe foliar chlorosis and severe stem lesions with no die back, and 5 = defoliation, severe stem lesions and die back (Gondwe et al., 2003). Cassava mosaic disease was also assessed at three (CMD3S) and six (CMD6S) after planting using a scale of 1 to 5; where 1 = no visible disease symptoms, 2 = mild chlorotic pattern on entire leaflets or mild distortion at base of leaflets, rest of leaflets appearing green and healthy, 3 = strong mosaic pattern on entire leaf, and narrowing and distortion of lower one-third of leaflets, 4 = severe mosaic, distortion of two-thirds of leaflets and general reduction of leaf size, and 5 = severe mosaic, distortion of four-fifths or more of leaflets, twisted and misshapen leaves (IITA, 1990).

At twelve months after planting, clonal trials were harvested to allow evaluation of fresh root weight (FRW) and cassava brown streak root necrosis severity (CBSDRS). All the ten plants were harvested and partitioned into roots and above-ground biomass (leaves and stems). Fresh root weight (FRW) and above-ground biomass were separately weighed (kg plot^−1^) using a hanging weighing scale of 200 kg capacity. On the other hand, CBSDRS was recorded on all harvested roots per plot using a scale of 1-5; where 1 = no observable necrosis, 2 = ≤ 5% of root necrotic, 3 = 6 to 25% of root necrotic, 4 = 26 to 50% of root necrotic with mild root constriction, and 5 = > 50% of root necrosis with severe root constriction (Gondwe et al., 2003).

The validation set comprised germplasm that was sourced from National Roots Crops Research Institute (NRCRI), Nigeria. A total of 5,000 botanical seeds were generated from bi-parental crosses involving forty-eight elite progenitors. The progenitors were selected per se based on their yielding ability and resistance to cassava mosaic disease (CMD). Accordingly, these seeds were shipped and planted in a seedling nursery at Namulonge. Out of the 5000 botanical seeds, 1980 successfully emerged, giving rise to 106 families. The 1980 seedlings were thus established in an unreplicated seedling trial during the second rains of 2018 (September/October). A total of 569 clones were selected from the seedling trial for further evaluation at the clonal stage during the 2019-2020 season, which was laid out using an augmented randomized block design at Namulonge. At the end of clonal evaluation, only 297 clones remained, as half of the clones were directly culled by CBSD. These 297 clones constituted the validation set for genomic prediction, and were assessed for CMD and CBSD severity fresh root yield as the 3rd trait evaluated, following the same procedure previously described for the training population.

### Genotyping of the training and validation sets

Leaf samples were obtained from the clonal evaluation stage of the training (C0, C1, C2) and the validation (germplasm from Nigeria) populations and shipped to Intertek, Australia, for DNA extraction and genotyping. Both C0 and C1 clones were originally genotyped using genotyping by sequencing (GBS) platform with 46K single nucleotide polymorphism (SNP) chip at Genomic Diversity facility of Cornell University (Ozimati et al., 2018). However, because the National Cassava Breeding Program of Uganda recently opted for Diversity Arrays Technology (DArT) genotyping services for routine genomic selection work, SNP markers from GBS (for both C0 and C1) were later imputed with those from DArT platform, giving rise to 23K SNP markers for genomic selection. Genotyping of the C2 and validation population (germplasm from Nigeria) was therefore done by the DArT platform, Australia, using the same 23K SNP markers that had been used previously to genotype C0 and C1 populations. Missing markers of the genotyped individuals were filled in by imputation, using markers from the East Africa imputation reference panel using BEAGLE software version 5.0 (Browning and Browning, 2007). The markers were thereafter filtered, and those with minor allele frequency (MAF) greater than 0.01 (21,938 SNPs) were used for downstream analyses.

We used the 21,938 SNPs to assess the population structure of the training population from Uganda (C0, C1 and C2) and validation population from West Africa. The SNP genotypes were coded as -1, 0, or +1. Principal component analysis (PCA) was done on scaled SNP markers using the *prcomp* function in R. The first two principal components (PC) were used to visualize population structure.

### Estimates of broad-sense heritability, genetic gain and accuracy of genomic prediction

To estimate heritability for each trait per cycle of GS population (C0, C1 and C2) and the WA clones, we fitted linear mixed models based on experimental design for each trial, followed by extraction of variance components using restricted maximum likelihood procedure (Spilke et al., 2005). The variance components were then used for estimation of broad-sense heritability per trait. Because C0 trial from Kasese in 2014 generally had low broad-sense heritability estimates across traits, the trial was not included for subsequent genomic prediction analyses.

For genomic prediction, we fitted a two-stage prediction model. At the first stage the raw phenotypes were merged across trials (training [C0, C1 and C2] and validation trial [WA]) into a single data set and fitted the linear mixed model using *lme4* package in R, accounting for the environmental differences as well as trial evaluation year as below:


$$\;y=\;\text{X}\beta \;+\;{\text{Z}}_{clone}c+{\text{Z}}_{rep(loc/study\;year)}r+\text{ϵ}$$

where, *y* represents raw phenotypic value; *β* represents fixed effect of the grand population mean, (C0, C1, C2 and WA), study year, and location, with ***X*** being the corresponding incidence matrix linking observations to those effects. *c* and *r* represent random effects of clones with $c∼N(0,I\sigma_{c}^{2})$ , and replication nested in location-study year such that $r∼N(0,I\sigma_{r}^{2})$ with ***Z*** *_clone_* and ***Z*** *_rep_*_(_ *_loc/study year_*_)_ being corresponding incidence matrices for clones and replications nested in location-study year respectively. The residuals ϵ were distributed as: $\text{ϵ}∼N(0,\;I\sigma_{\text{ϵ}}^{2})$ with I representing the identity matrix. We extracted best linear unbiased predictors (BLUPs) for each clone using the *ranef* function available in *lme4* package (Bates et al., 2015), and these were preferred over fixed clone effects for the genomic prediction study due to imbalances in the dataset.

After extraction of BLUPs for each clone for each cycle, we fitted a G-BLUP model to estimate genomic estimated breeding values (GEBVs) that were used for assessment of gains from genomic selection using the three evaluated Uganda’s GS cycles (C0, C1 and C2). A one-way analysis of variance was performed to test for significant differences among the means of the GEBVs for the three cycles for each trait using R (R Core Team, 2021). Mean GEBVs of three cycles were separated using Tukey’s honestly significant difference. Gains from genomic selection were thereafter calculated as the difference between the mean performance of new cycle and mean performance of the previous cycle from which the new cycle was selected.

Furthermore, we carried out 5-fold cross-validation analyses for each training population (C0, C1 and C2) and WA clones. To do the cross-validation, the BLUPs that were extracted from the first stage analyses per trait were used as the response variable to fit a second stage prediction model for five genomic prediction models with different statistical assumptions. These models were: genomic best linear unbiased prediction (G-BLUP) (VanRaden, 2008; Endelman, 2011), Bayesian ridge regression BRR (Meuwissen et al., 2001), Bayesian least absolute shrinkage and selection operator (BL), Bayes A and Bayes B (Park and Casella, 2008). An excellent review of these models has already been provided by Heslot et al. (2012), and thus will be discussed briefly.

To implement G-BLUP, we fitted a model: *Y* = 1*β* +*Xg* + ϵ , with *g* ~ N (0, K*σ^2^*_g_) and ϵ ~ (0, I*σ^2^*_g_), where Y represents the vector of BLUPs, *β* represents an overall population mean, X represents the design matrix linking observations to genomic values, *g* being vector of genomic estimated breeding values for each clone, and ϵ represents the vector of residuals. We assumed, *g* has a known covariance structure defined by the realized genomic relationship matrix K, while I representing identity matrix.

Additional, we implemented the four Bayesian models i.e. BRR, BL, Bayes A and Bayes B, following the same linear mixed model: *Y* = 1*β* +*Zg* + ϵ , with *g*~N (0, K*σ^2^*_g_) and ϵ ~ (0 I*σ^2^*_g_), where Y represents the vector of BLUPs, *β* represents an overall population mean, X represents the design matrix linking observations to genomic values, *g* being vector of genomic estimated breeding values for each clone, and ϵ represents the vector of residuals. We assumed, *g* also has a known covariance structure defined by the realized genomic relationship matrix K and I representing identity matrix. Specifically, BRR assigns a Gaussian prior with common variance to each marker effect, and applies homogeneous shrinkage to all marker effects. BL employs a double-exponential prior distribution for marker effects, which places strong shrinkage to markers with little to no effect on the trait. Bayes A applies a scaled-t prior distribution to marker effects, and places slightly less shrinkage on markers with zero effect, thereby allowing more flexibility for marker effects. Lastly, Bayes B assumes that most of the markers have zero effect on the trait, and assumes that the markers with an effect on the trait will follow a scaled-t prior distribution as in the case of Bayes A, making it relatively more stringent when compared to Bayes A. All the four Bayesian models used in this study were fitted using the *BGLR* function available in the R package *BGLR* (Pérez and de los Campos, 2014). A Markov Chain Monte Carlo (MCMC) algorithm was applied with prior parameters defined following the procedure suggested by de los Campos et al. (2013). Computations were performed using a chain length of 10,000 iterations, with the first 1000 iterations discarded as burn-in (Pérez and de los Campos, 2014).

Briefly, during implementation of cross-validation within each population (C0, C1, C2 and validation), the clones were randomly split into five subsets (5-fold), where 4/5 of the subsets were used to train the model, while 1/5 was reserved for model validation and this was replicated 5 times. The accuracy of genomic prediction for each fold was then computed as Pearson correlation coefficient between the genomic estimated breeding values and BLUPs for each trait as a response variable.

Lastly, we carried out independent validation for the WA clones, the five evaluated genomic prediction models (G-BLUP, BRR, BL, Bayes A, and Bayes B) were trained using C_0,_ C1 and C2 to predict disease severity and fresh root weight in the validation population (West African population) that comprised 297 clones. Similarly, the prediction accuracy for each model was assessed using Pearson’s correlation between the GEBVs and the BLUP values per trait.

## Results

### Broad sense heritability for evaluated traits in the training and validation populations

Plot-based heritabilities were low to intermediate (**Table 1**). Estimates of plot-based broad-sense heritability for the training set were highest for disease traits, and these ranged from 0.04 to 0.99 for CBSD foliar severity, 0.2 to 0.86 for CBSD root necrosis severity and 0.00 to 0.99 for CMD severity. Differences in trait heritabilities for data collected at two time points (three and six months after planting) were not substantial, for both CBSD and CMD severity. Heritabilities for fresh root weight were generally modest, ranging from 0.00 to 0.99. Lowest heritabilities for disease traits were observed at Kasese in the mid-western Uganda, while highest heritability for both disease severity and fresh root weight was observed at Serere in Eastern Uganda. Namulonge (central Uganda) registered the lowest heritability for fresh root weight. Though heritability for fresh root weight in the validation population was 0.00, heritabilities for CBSD and CMD severity were moderately high (H^2^ > 0.65).

**Table 1:** Plot based broad sense heritability estimates for disease severity and fresh root weight for training and validation populations evaluated at the different locations in Uganda between 2013 to 2019.

Population	Year	Location	CBSD3S	CBSD6S	CBSDRS	CMD3S	CMD6S	FRW
C0	2013	Kasese	0.31	0.30	0.45	0.64	0.45	0.40
C0	2013	Namulonge	0.33	0.37	0.60	0.42	0.74	0.40
C0	2013	Ngetta	–	0.52	0.68	0.75	–	0.47
C0	2014	Kasese	0.04	0.06	–	0.00	0.00	–
C0	2014	Namulonge	0.38	0.37	0.68	0.49	0.77	–
C1	2016	Namulonge	0.40	0.17	0.20	0.80	0.83	0.57
C1	2016	Serere	0.65	0.46	0.66	0.80	0.79	0.02
C1	2017	Namulonge	0.55	0.45	0.44	0.79	0.71	0.11
C1	2017	Serere	0.99	0.99	0.86	0.99	0.94	0.99
C2	2019	Namulonge	0.70	0.54	0.5	0.84	0.90	0.00
WA	2019	Namulonge	0.81	0.67	0.84	0.91	0.94	0.00

CBSD3S and CBSD6S, cassava brown streak disease foliar severity at 3 and 6 months after planting respectively; and CBSDRS, cassava brown streak disease root severity at 12 months after planting.

### Gains from genomic selection in Uganda’s breeding populations from 2013 to 2019

**Figure 1:**
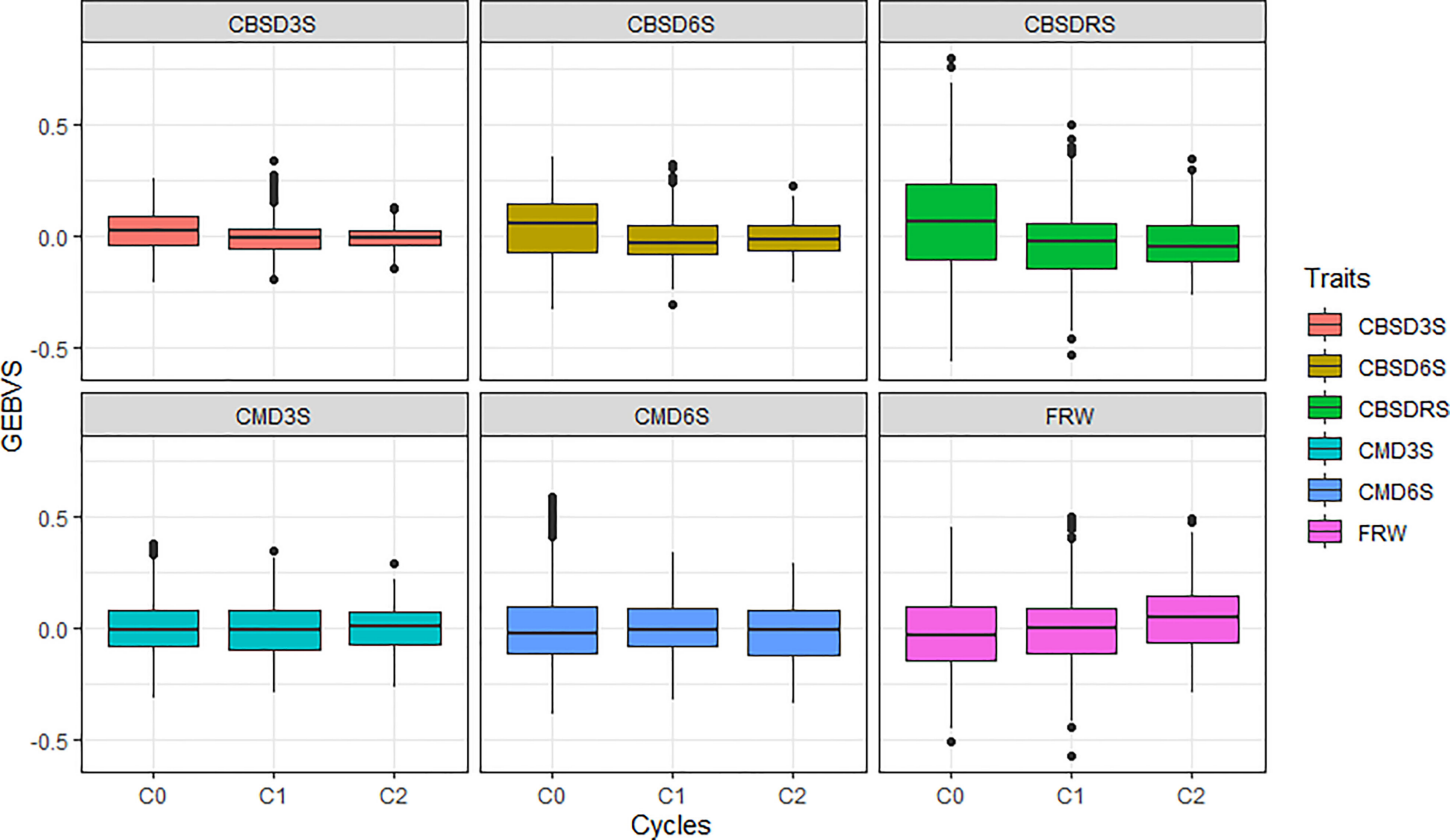
Performance of the three cycles (C0, C1 and C2) of Uganda’s cassava genomic selection population for disease resistance. CBSD3S, cassava brown streak disease foliar severity scored at three months; CBSD6S, Cassava brown streak disease foliar severity scored at six months; CBSDRS, Cassava brown streak disease root severity scored at 12 months; CMD3S, Cassava mosaic disease severity scored at three months; CMD6S, Cassava mosaic disease severity scored at six months.

Using a boxplot, we summarized variations for genomic estimated breeding values across GS cycles for the six traits assessed, with overall genetic progress recorded for most traits except for CMD (**Figure 1**). Based on average genomic estimated breeding values per cycle (GEBVs), CBSD foliar severity at three months reduced from a mean GEBV of 0.016 for C0 to -0.008 in C1. CBSD foliar severity at six months and CBSD root necrosis severity exhibited a similar downward trend in disease severity when C0 clones where recombined and advanced to C1 using genomic selection (**Table 2**). With regard to CMD severity, mean GEBVs reduced from 0.006 to 0.004, and 0.013 to -0.002, for CMD3S and CMD6S, respectively, as clones were advanced from C0 to C1. Fresh root weight also increased from -0.017 in C0 to -0.004 to C1. From C1 to C2, all disease traits i.e. CBSD3, CBSD6S, CBSDRS, CMD3S and CMD6S further exhibited a downward trend in disease severity based on their mean GEBVs. Fresh root weight also continued to exhibit an upward trend when C1 clones were recombined and advanced to C2 of genomic selection. Highest response to selection was observed with fresh root weight, CBSD root necrosis resistance, fresh root weight, CBSD foliar severity, and lastly CMD severity.

**Table 2:** Mean performance of genomic selection cycles and corresponding gains from selection for fresh root weight and virus disease resistance.

Cycle	FRW	CBSD3S	CBSD6S	CBSDRS	CMD3S	CMD6S
C_0_	-0.017^a^	0.016^a^	0.034^a^	0.063^a^	0.006^a^	0.013^a^
C_1_	-0.004^a^	-0.008^b^	-0.019^b^	-0.031^b^	-0.004^a^	-0.002^a^
C_2_	0.044^b^	-0.009^b^	-0.013^b^	-0.034^b^	-0.001^a^	-0.017^b^
P-value	***	***	***	***	NS	*
*Gains from selection*						
C_1_ - C_0_	0.013	-0.024	-0.054	-0.094	-0.009	-0.015
C_2_ - C_1_	0.048	-0.001	0.006	-0.003	0.003	-0.014

CBSD3S, cassava brown streak disease foliar severity scored at three months; CBSD6S, Cassava brown streak disease foliar severity scored at six months; CBSDRS, Cassava brown streak disease root severity scored at 12 months; CMD3S, Cassava mosaic disease severity scored at three months; CMD6S, Cassava mosaic disease severity scored at six months; and FRW, fresh root weight. Letters indicate significant differences using Tukey’s honestly significant difference (α = 0.05). * P < 0.05, *** P < 0.001, and NS = non-significant differences between average performance of selection cycles.

### Population structure between training and validation sets

**Figure 2:**
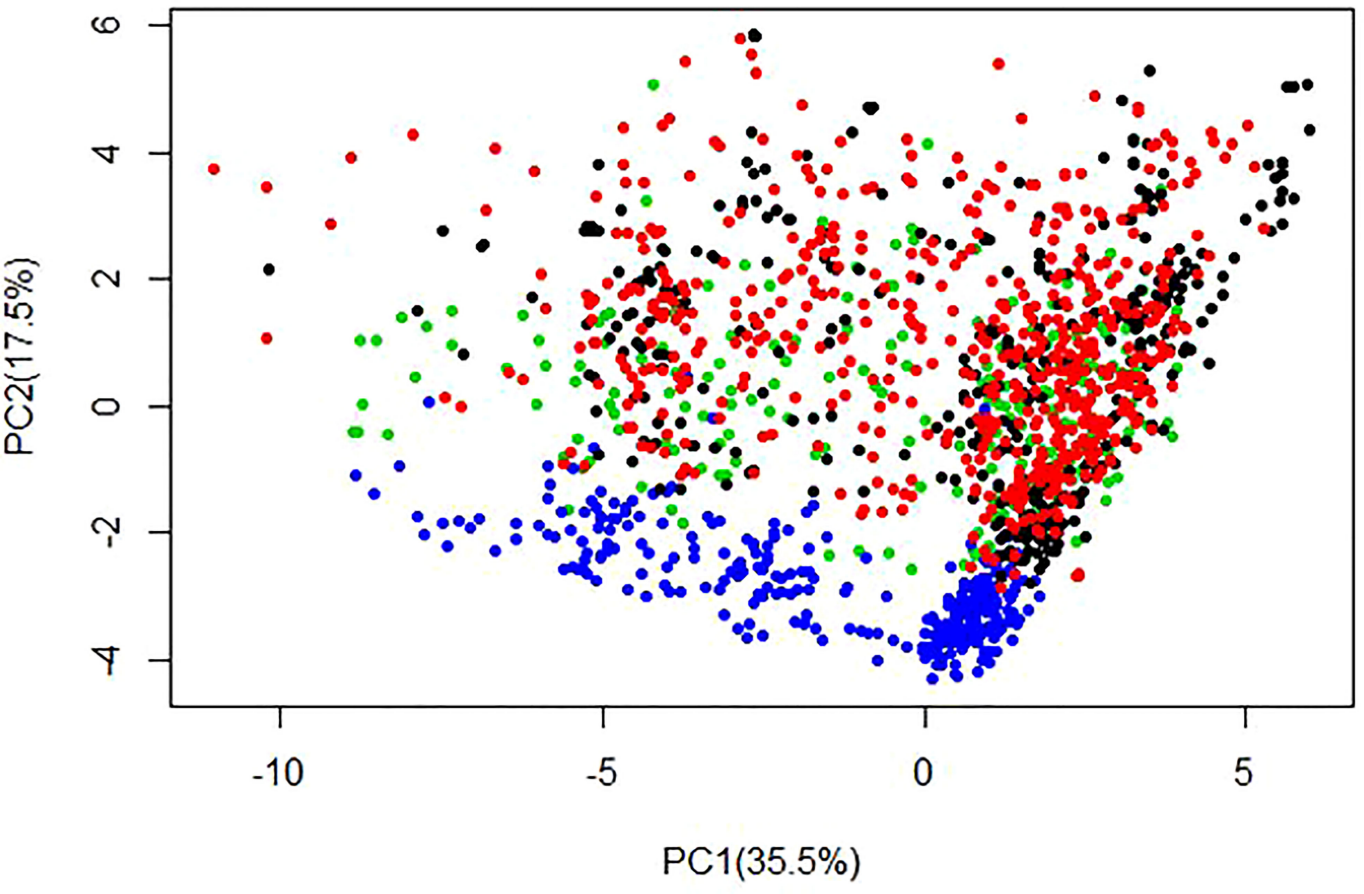
Population structure displayed by the first two principal components (PCs) generated for training set i.e. C0 (384 clones), C1 (638 clones), C2 (287 clones) and the validation population (279 clones from West Africa) using 21,938 SNP markers. The figure displays population structure from PC1 vs PC2, with associated variances for each PC represented in brackets. Black = C0, Red = C1, Green = C2 and Blue = WA clones.

Principal component analysis revealed a slight genetic differentiation between the Ugandan and West African cassava populations (**Figure 2**). Variations in genetic structure between the Ugandan and West African populations were moderate, as the first two principal components (PCs) explained approximately 53% of the variation, where the first and second PCs accounted for 35.5%, and 17.5%, respectively.

### Cross-validation prediction accuracies within the training and validation populations

**Figure 3:**
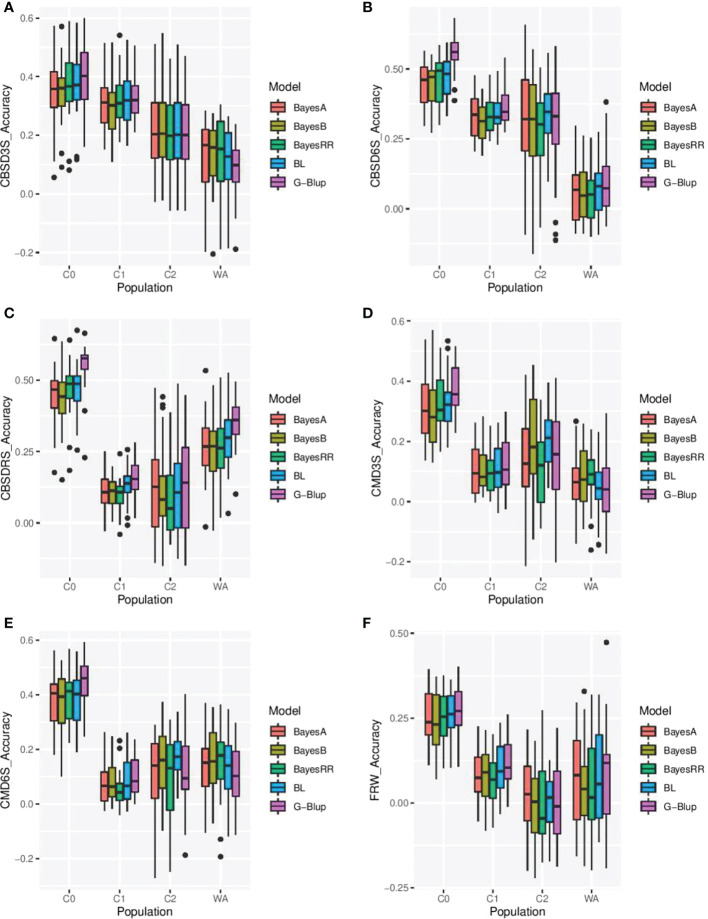
Cross validation prediction accuracies for cassava brown streak disease severity at three (CBSD3S), six (CBSD6S) and twelve months after planting (CBSDRS), cassava mosaic disease severity at three months (CMD3S) and six months after planting (CMD6S), and fresh root weight (FRW) using five genomic prediction models in the training (C0, C1, C2) and validation population (germplasm from West Africa). **(A–F)** Represent prediction accuracies for CBSD3S, CBSD6S, CBSDRS, CMD3S, CMD6S and FRW, respectively. C0, C1, C2 represent cycle zero, cycle one, cycle two of Uganda’s cassava genomic selection population, while WA represent West African cassava germplasm from Nigeria. BL, Bayesian Least Absolute shrinkage and selection operator, BRR, Bayesian Ridge Regression, and G-Blup, Genomic Best Linear Unbiased Prediction.

Cross-validation prediction accuracies were performed using five models (Bayes A, Bayes B, BRR, BL, and G BLUP) to assess prediction accuracy of genomic selection for CBSD resistance, CMD resistance and fresh root weight within the training and validation populations. We observed modest prediction accuracies for all evaluated traits and populations (**Figure 3**). Prediction accuracies in the training set ranged from -0.06 to 0.59 for CBSD3S, -0.16 to 0.68 for CBSD6S, -0.15 to 0.68 for CBSDRS, -0.21 to 0.57 for CMD3S, -0.27 to 0.59 for CMD6S, and -0.22 to 0.41for FRW. Of the three cycles in the training set, C0 registered the highest prediction accuracies for all traits, followed by C1 and lastly C2. Average prediction accuracies for C0 were: 0.37, 0.48, 0.48, 0.33, 0.40 and 0.26 for CBSD3S, CBSD6S, CBSDRS, CMD3S, CMD6S and FRW, respectively. Average prediction accuracies for C1 were: 0.32, 0.34, 0.12, 0.11, 0.08 and 0.08, for CBSD3S, CBSD6S, CBSDRS, CMD3S, CMD6S and FRW, respectively. Lastly, mean prediction accuracies for C2 were: 0.21, 0.30, 0.11, 0.16, 0.13 and 0.00 for CBSD3S, CBSD6S, CBSDRS, CMD3S, CMD6S and FRW, respectively. Across the three cycles and the evaluated models, CBSD6S was predicted with the highest accuracy (0.37), followed by CBSD3S (0.29), CBSDRS (0.22) and lastly fresh root weight (0.11). We observed that GBLUP was slightly superior to all evaluated Bayesians models across the five traits and three populations in the training set. On the other hand, cross-validation predictions in the validation set (clones from West Africa) were relatively lower than those observed in the training population (clones from Uganda). Prediction accuracies ranged from -0.25 to 0.31 for CBSD3S, -0.09 to 0.38 for CBSD6S, -0.36 to 0.53 for CBSDRS, -0.17 to 0.29 for CMD3S, -0.19 to 0.37 for CMD6S, and -0.10 to 0.47 for FRW. On average, CBSDRS was predicted with the highest accuracy (0.29), followed by CMD6S (0.13) and lastly FRW (0.07).

### Using Uganda’s training population to predict traits in West African clones

Analyses were performed using C0, C1, and C2 to assess prediction accuracy of genomic selection for CBSD resistance, CMD resistance and FRW in the West African population (297 clones) that was part of the pre-breeding populations evaluated in Uganda for CBSD resistance. We observed extremely low prediction accuracies for all traits (**Table 3**). For example, prediction accuracies ranged from -0.07 to 0.10 for CBSD3S, -0.02 to 0.15 for CBSD6S, -0.18 to 0.05 for CBSDRS, 0.002 to 0.09 for CMD3S, -0.034 to 0.078 for CMD6S and lastly -0.076 to 0.086 for FRW. Average predictions were less than 0.1 for all evaluated traits. Though predictions were extremely low, C1 registered the highest prediction for CBSD6S (0.14) in WA population, and lowest prediction was observed when C0 was used to predict CBSDRS in the validation set. Since prediction accuracies were extremely low, it seemed unreasonable to assess how a combination of the three populations would affect prediction accuracies of GS in the WA population.

**Table 3:** Independent validation prediction accuracies for cassava mosaic disease severity, cassava brown streak disease severity and fresh root weight using five genomic prediction models and three cycles of genomic selection.

Cycle	Model	CBSD3S	CBSD6S	CBSDRS	CMD3S	CMD6S	FRW
C0	BayesB	-0.057	-0.019	-0.085	0.007	0.032	0.007
C0	BayesA	-0.056	-0.011	-0.061	0.042	0.068	0.042
C0	BL	-0.057	-0.008	-0.084	0.074	0.078	0.074
C0	G-Blup	-0.039	0.019	-0.181	0.057	0.075	0.057
C0	BayesRR	-0.072	-0.007	-0.085	0.086	0.073	0.086
C1	BayesB	0.066	0.151	-0.086	0.036	0.044	-0.028
C1	BayesA	0.093	0.153	-0.151	0.002	0.014	-0.013
C1	BL	0.085	0.153	-0.053	0.042	0.043	-0.015
C1	G-Blup	0.088	0.107	-0.072	0.005	0.002	-0.022
C1	BayesRR	0.104	0.154	-0.089	0.037	0.066	0.012
C2	BayesB	0.028	0.124	0.056	0.032	0.021	-0.006
C2	BayesA	0.053	0.125	-0.016	0.052	-0.034	-0.017
C2	BL	0.052	0.141	0.012	0.092	-0.022	-0.076
C2	G-Blup	0.039	0.096	0.021	0.049	-0.010	-0.069
C2	BayesRR	0.017	0.131	-0.003	0.058	-0.010	-0.034

BL, Bayesian least absolute shrinkage and selection operator; G-BLUP, Genomic best linear unbiased prediction method; BRR, Bayesian Ridge Regression. CBSD3S, cassava brown streak disease foliar severity scored at three months; CBSD6S, Cassava brown streak disease foliar severity scored at six months; CBSDRS, Cassava brown streak disease root severity scored at 12 months; CMD3S, Cassava mosaic disease severity scored at three months; CMD6S, Cassava mosaic disease severity scored at six months; and FRW, fresh root weight.

## Discussion

The challenges of rapid human population growth and climate change invariably affect agricultural productivity, and thus the need for increased genetic gains (Hickey et al., 2017). Currently, there are concerted global efforts to combat CBSD, a disease that is endemic to East and Central Africa but posing a significant threat to cassava production in West Africa, the world’s largest producer and consumer of cassava (Legg et al., 2014). In this study, we leveraged genomic prediction approaches as a possible means to enable pre-emptive breeding for CBSD resistance in West Africa, using elite cassava populations from Uganda. Accordingly, three Uganda’s populations segregating for CBSD severity comprised the training set, and these were used to predict CBSD resistance along with other equally important traits such as CMD resistance and fresh root weight in the WA population that was evaluated in Uganda, a hotspot for CBSD.

Broad sense heritability estimates for evaluated traits were low (H^2^< 0.2) to high (H^2^ > 0.6), and were well in range with heritability estimates in literature (Kayondo et al., 2018; Okul et al., 2018; Ozimati et al., 2018). These results underpin the general conclusion that the experimental sites were hotspots for CMD and CBSD i.e. the disease pressure was high enough to cause substantial variation in clone response to the virus diseases (Alicai et al., 2019). This finding further implies that Namulonge and Serere are suitable for screening of germplasm against CMD and CBSD, and could be used by breeding programs threatened by CBSD. The extremely low heritability estimates for CMD severity are attributable to low phenotypic variations for CMD in the evaluated Ugandan cassava populations. The low phenotypic variations for CMD resistance traits were attributable to the fact that breeding efforts targeting resistance to CMD have been ongoing since 1930s (Legg and Thresh, 2000), which is sufficient time for increasing the frequency of resistance alleles in the breeding populations (Hallauer et al., 1988), and thus we might have fixed CMD resistance alleles in our recently developed cassava germplasm. The low heritabilities of CBSD traits are also attributable to the low phenotypic variations in CBSD severity observed in C0, C1 and C2, which were also attributable to selection and recombination. These low phenotypic variations for CMD and CBSD resistance imply that the breeding program has attained a usable level of resistance to virus diseases in most of its elite material, and therefore, alleles for yield and end-user preferred traits need to be introgressed into disease resistance background to allow enhancement of yield traits.

We observed substantial gains for all evaluated traits in Uganda’s GS cycles. This finding is agreement with findings from Sweeney et al. (2021) who reported increased gains from GS in the spring barley breeding program. The observed improvements in trait means based on their GEBVs is an indication that genomic selection successfully increased frequency of desirable alleles for target traits in the evaluated cassava populations. These findings further imply that even with low predictions accuracies of less than 0.40, genetic gains are possible with GS for low heritability traits. The low gains in CMD resistance could be due to low phenotypic variability in the evaluated traits i.e. clones exhibited a similar level of resistance both at three and six months after planting for the three evaluated cycles with a mean severity score of 1.4. With the observed downward trend in disease severity and a concurrent upward trend in fresh root weight, genomic selection is likely to fast-track variety replacement and/or increase variety turnover in cassava especially in this era of climate change and rapid population increase.

Having observed significant gains in traits using genomic selection, we evaluated the importance of our GS cassava populations in predicting cassava traits in West Africa, where CBSD is an eminent threat. Based on principal component analysis of SNP data, we observed a close relationship between the training set (Uganda’s cassava populations) and validation set (West African cassava population), with a slight population structure and genetic differentiation between the two populations (**Figure 2**). This low genetic variability and lack of clear structure within these populations underpins the likelihood that the East and West African materials might have shared a common ancestry, a situation that could be attributed to germplasm exchange between east and west Africa in the 1930s, during the advent of cassava mosaic disease (CMD) pandemic (Jennings, 2002). The absence of clear population structure and low genetic variation between the evaluated populations also suggested the appropriateness of using Uganda’s population (C0, C1, and C2) as a training population for genomic prediction of the West African populations and subsequent selection of individuals using GEBVs as a pre-emptive breeding strategy. Accordingly, analyses were performed to determine whether the close relationship between the populations would result into high prediction accuracies when Uganda’s population was used to train models for prediction of disease resistance and fresh root weight in the WA population. Surprisingly, the west African population which was fairly genetically similar to Uganda’s training population, was predicted with extremely low accuracy (ranging from -0.07 to 0.15) for all evaluated traits when C0, C1 and C2 were separately used as training populations, suggesting that there could be other factors that affected the prediction accuracy of GS other than the relationship between training and validation sets.

Several genomic prediction models have been developed to predict trait performance under different genetic architecture and the five GP models (Bayes A, Bayes B, BRR, BL and G-BLUP) chosen for this study also differed in assumptions about the genetic architecture of the evaluated traits. Results revealed that models performed similarly for the most part, but there also occasions where G-BLUP was slightly superior to Bayesian models used in this study. These results were in good agreement with earlier findings from Wolfe et al. (2016) and Kayondo et al. (2018). Superiority of G-BLUP could be that the true QTL effects for evaluated traits were relatively small and that the distribution of these effects could be less extreme. The superiority of G-BLUP could be also attributed to its ability to take advantage of the relationships among individuals at the causal loci for the traits under analysis (VanRaden, 2008), indicating that models that might estimate relationship information between training and test sets could be more valuable than those that estimate marker effects directly.

Average cross validation prediction accuracies across the three populations for CBSD and CMD resistance fresh root weight ranged between 0.26 to 0.48, and were comparable to findings by Wolfe et al. (2017); Wolfe et al. (2016); Kayondo et al. (2018) and Ozimati et al. (2018). The low cross validation prediction accuracies suggested that they could be attributed to the low phenotypic variations for the studied traits observed in the evaluated populations. These cross validation predictions within the three populations were encouraging and thus highlighting the utility of GS for improving CBSD and CMD resistance, and fresh root weight. On the other hand, cross validation prediction accuracies in the validation set (west African clones) were much lower than that was observed in the Ugandan training set, and this could be attributed to the fact that west African clones might be deficient in CBSD resistance alleles (Ano et al., 2021).

On the other hand, independent validation prediction accuracies of genomic selection were generally low, and they were lower than cross-validation prediction accuracies for CMD, CBSD traits and fresh root weight. Given that the training and validation populations were fairly genetically similar, the low prediction accuracies for independent validations could be attributed to genotype by environment interaction i.e. the training and validation populations were evaluated during different seasons. These low predictions could also be attributed to the fact that west African cassava populations were deficient in CBSD resistance alleles (Ano et al., 2021). No consistent superior performance was observed for any of the prediction models that were assessed, and this was in good agreement with Heslot et al. (2012) and Jannink et al. (2010). Although the models tested in this study assumed different distributions of marker effects (Meuwissen et al., 2001; Lorenz et al., 2011), their similarity in prediction accuracies could be interpreted as approximation to optimal genomic prediction models, where all the models capture the same or similar QTL effects across the genome (Su et al., 2014). In such a situation, choice of GS model would be less important than choice of training population.

The C0 training set yielded the lowest prediction accuracies, with negative average accuracies for all traits across all models. The disparity between the predictive ability of the C1, C2 and the C0 training sets might be because the C1 training set was able to capture more genetic signals for CBSD foliar and root symptom expression in the West African clones than C0 training set. This phenomenon was noted by Ozimati et al. (2018) who reported that optimized Ugandan training sets were able to capture more genetic signals and yielded higher prediction accuracies for CBSD resistance in IITA clones than random training sets. Another possible explanation might be that the quantitative trait loci (QTLs) responsible for CBSD resistance in the two populations were different. This might be due to recombination events that might have occurred in their genomes, resulting in the rearrangement of QTLs responsible for CBSD resistance in the three populations.

## Conclusion

Based on the findings of this study, the breeding program in Uganda has made genetic progress through GS accelerated breeding cycles for most target traits, especially for CBSD root necrosis which is one of the must to have traits in a variety, demonstrating the worthwhile of GS for rapid population improvement and variety development. In general, low prediction accuracies were recorded from using Ugandan training set to predict traits in African clones, suggesting inadequacy of utilizing Ugandan training set, especially for CBSD pre-emptive breeding in WA. In this case, efforts should be devoted to sharing Uganda’s germplasm that possess resistance with the W. African breeding programs for hybridization to fully enable deployment of genomic selection as a pre-emptive CBSD breeding strategy in W.A

## Data availability statement

Publicly available datasets were analyzed in this study. This data can be found here: https://www.nextgencassava.org.

## Author contributions

OA Conceptualized the study, collected and analyzed data, and wrote the original manuscript. WE collected data and reviewed the manuscript. AU collected data and reviewed the manuscript. FM collected and analyzed the data, and reviewed the manuscript. PI collected data and reviewed the manuscript. MK collected data and reviewed the manuscript. CE acquisition of the funding and reviewed the manuscript. RK collected data and reviewed the manuscript. All authors contributed to the article and approved the submitted version.

## Funding

This work was supported by Next Generation Cassava Breeding Project funded by Bill and Melinda Gates Foundation, funding granted through Cornell University (grant number OPP1048542).

## Conflict of interest

The authors declare that the research was conducted in the absence of any commercial or financial relationships that could be construed as a potential conflict of interest.

## Publisher’s note

All claims expressed in this article are solely those of the authors and do not necessarily represent those of their affiliated organizations, or those of the publisher, the editors and the reviewers. Any product that may be evaluated in this article, or claim that may be made by its manufacturer, is not guaranteed or endorsed by the publisher.
